# Serum Vaspin Concentration in Elderly Type 2 Diabetes Mellitus Patients with Differing Body Mass Index: A Cross-Sectional Study

**DOI:** 10.1155/2017/4875026

**Published:** 2017-05-03

**Authors:** Wei Yang, Yun Li, Tian Tian, Li Wang

**Affiliations:** ^1^Department of Geriatric Medicine, Capital Medical University, Xuan Wu Hospital, Beijing 100053, China; ^2^Department of Endocrine, Capital Medical University, Xuan Wu Hospital, Beijing 100053, China

## Abstract

*Aims*. This study was to evaluate the association of serum vaspin concentrations with body mass index (BMI) among elderly patients (>60 years old).* Methods*. A total of 227 elderly individuals included 76 healthy with normal glucose tolerance, which divided into normal weight control (BMI < 25, *n* = 38) and overweight or obese control (BMI ≥ 25, *n* = 38) subgroups, and 150 T2DM patients, which divided into normal weight diabetes (BMI < 25, *n* = 55), overweight diabetes (30 > BMI ≥ 25, *n* = 52), and obese diabetes (BMI ≥ 30, *n* = 43) subgroups. Relevant parameters were matched for age and gender ratio. Serum vaspin concentrations were measured by enzyme-linked immunosorbent assay.* Results*. Serum vaspin concentration was significantly higher in the T2DM than the healthy (451.9 ± 32.6 versus 284.2 ± 21.7, *P* < 0.01). In the diabetic patients, the vaspin concentration was significantly higher in the obese group than the normal weight group (498.2 ± 17.1 versus 382.1 ± 21.3, *P* < 0.05). In addition, the concentration of vaspin in normal weight T2DM was higher than in healthy control group with normal weight (382.1 ± 21.3 versus 192.5 ± 45.2, *P* < 0.05). Multiple regression analysis revealed that BMI was independent factors influencing the serum vaspin concentration in all participants.* Conclusion*. Vaspin may play an important compensatory role in obesity and insulin resistance in elderly people. The clinical trial registration number is ChiCTR-OPC-14005698.

## 1. Introduction 

In recent years, the incidence of obesity, especially among older adults, has continued to increase [1]. Elderly adults, due to their special metabolic characteristics, have an increased risk of suffering from obesity, especially abdominal obesity. The link between obesity and adverse health outcomes such as diabetes, cancer, and cardiovascular disease has been well established, and, thus, obesity poses a major challenge in current and future health care provision [2]. In particular, older people with type 2 diabetes mellitus (T2DM) will be a focus of geriatric medical research.

At present, fat tissue is considered to be an endocrine gland that produces bioactive adipokines, which take part in glucose and lipid metabolism [3]. Increased visceral adipose tissue mass is associated with a higher prevalence of insulin resistance and risks of T2DM and cardiovascular diseases. Adipokines take part in glucose and lipid metabolism as well as the body's immune response and are often the cause of obesity-related diseases [4].

Vaspin (visceral adipose tissue-derived serine protease inhibitor), as a novel adipocytokines, was isolated from the visceral adipose tissue of spontaneously obese Otsuka Long-Evans Tokushima Fatty rats with T2DM in 2005 [5]. Vaspin has been reported as a candidate linking human obesity to its related metabolic alterations [6]. Previous research showed that vaspin was associated with obesity and glucose metabolism, and administration of vaspin in obese mice improved glucose tolerance and insulin sensitivity and reduced food intake [7]. Some reports investigating serum vaspin levels in T2DM patients [8, 9] were available. Most published reports were from Caucasians in Europe, non-Chinese people, Japanese [8, 10], and Egyptians [9]. There have been few studies investigating the serum vaspin levels in Chinese elderly people, likely due to the heterogeneity of the elderly population and because older patients often have multiple chronic diseases. In order to elucidate the relationships among the serum vaspin concentration, obesity, and T2DM in older patients, we designed this study to identify trends in the circulating vaspin concentration in Chinese elderly T2DM patients. We also explored the relation between vaspin and body mass index (BMI), age, gender, glucose, lipid metabolism, and insulin sensitivity among them.

## 2. Patients and Methods

### 2.1. Study Design

For this cross-sectional observational study, a total of 150 patients (>60 years of age) diagnosed with T2DM were enrolled from July 2014 to January 2016. Additionally, 76 individuals without T2DM were selected from the Department of Geriatrics and Endocrinology of Xuanwu Hospital to serve as a control group.

The research was approved by the hospital local ethics committee NO [2013]-001.

The control individuals are also divided into two groups according to BMI: the normal weight control (NWC) group (BMI < 25 kg/m^2^; *n* = 38 cases including 28 males and 10 females; mean age: 74.2 ± 4.2 years) and the obese control (OC) group (BMI ≥ 30 kg/m^2^; *n* = 38, including 27 males and 11 females; mean age: 75.2 ± 1.7 years) based on health criteria for obesity in WHO and NIH [11]. We first analyzed differences among the separate control groups and the T2DM patients as a whole group (T2DM group). Then, also according to BMI, the 150 elderly T2DM patients were divided into three subgroups: the normal weight diabetes (NWD) group (BMI < 25 kg/m^2^; *n* = 55, including 42 males and 13 females; mean age: 75.8 ± 2.2 years), the overweight diabetes (OWD) group (25 kg/m^2^⩽ BMI < 30 kg/m^2^; *n* = 52, including 38 males and 14 females; mean age: 78.5 ± 1.7 years), and the obese diabetes (OD) group (BMI ≥ 30 kg/m^2^; *n* = 43, including 31 males and 12 females; mean age: 75.2 ± 1.5 years). We then analyzed differences among the subgroups of T2DM patients and the control groups.

Healthy controls were recruited from the clinic of geriatric and endocrine department. All the participants underwent OGTT test and had confirmed normal glucose tolerance.

### 2.2. Basic Characteristics of T2DM

Among 150 diabetic patients, 25 were newly diagnosed when recruited and 125 had a diabetic duration of 16.26 ± 2.73 years. 15 patients were treated by therapeutic life intervention (diet and exercise), 50 patients took oral antidiabetic agents (5 cases with single agent, 20 cases with two agents, and 25 cases with three agents), 15 patients took intensive insulin therapy, and 70 patients were treated with a combined insulin and oral antidiabetic agents.

### 2.3. Inclusion and Exclusion Criteria

Patients who were >60 years and satisfied the standard diagnostic criteria for T2DM were included in the study. T2DM was diagnosed if they met one of three criteria as follows: (1) fasting plasma glucose (FPG) ⩾126 mg/dL, (2) random fasting glucose ⩾200 mg/dL in patient with symptoms of hyperglycemia, or (3) 2 h postprandial blood glucose level during an OGTT test ⩾200 mg/dL [12]. Patients were excluded if they had any of the following criteria: type 1 DM; acute complications of DM; severe infection; tumor history; any other endocrine disease or autoimmune disease; or use of hormonal preparations or immune inhibitors such as hydrocortisone, prednisone, methylprednisolone, cyclosporine A, and tacrolimus.

### 2.4. Anthropometric Measurements

The weight (kg) and height (cm) of all participants were recorded. BMI was calculated as body weight (kg) divided by the height squared (m^2^). Waist circumference (cm) was measured just above the uppermost lateral border of the right iliac crest. A horizontal mark was drawn and crossed with a vertical mark on the midaxillary line. The measuring tape was placed in a horizontal plane around the abdomen at the level of this marked point on the right side of the trunk.

Analysis of visceral adipose distribution was scanned by computed tomography (GE High Speed Advantage CT scanner, USA) at the umbilical level, and the area of visceral adipose was calculated.

### 2.5. Parameters for Clinical Assessment

After a 12 h overnight fast, blood samples were obtained from all subjects between 8 and 10 AM and immediately centrifuged. Aliquots of serum and plasma were taken for analysis of biochemical markers studied. Serum samples were stored at −80C until vaspin and insulin levels were measured. Fasting blood glucose (FBG) and 2 h postprandial glucose after 75 g oral glucose loading (2-h PBG) were measured by glucose oxidase method. Glycosylated hemoglobin (HbA1c %) was measured on a Cobas Integra 800 automated biochemistry analyzer (Roche, Basel, Switzerland).

Fasting serum insulin (FINS) concentration was measured by radioimmunoassay analysis method (Ray Bio, Norcross, GA). High-sensitivity C-reactive protein (hs-CRP) was detected by ELISA. Lipid profile, including total cholesterol (TC), triglyceride (TG), low-density lipoprotein (LDL-C), and high-density lipoprotein (HLD-C), were measured using a Hitachi 7600 automatic biochemical analyzer (Hitachi, Japan). All these were performed at the same time and site. Homeostasis model assessment of insulin resistance (HOMA-IR) as an insulin resistance index was measured using the equation: HOMA-IR = FPG (mmol/L) × FINS (*μ*U/L)/22.5. Based on the formula, coefficients of variation (CV) = (standard deviation/mean) × 100%, the inter- and intra-CV for insulin were calculated as 10% and 7%, respectively.

A portion of each blood sample was separated to obtain the serum by centrifugation at 3,500 rpm for five mins and kept at −80°C. The serum vaspin concentration was measured by ELISA (Shenzhen Lvshiyuan Biotechnology Co. Ltd., China) in a fully automatic multifunctional enzyme standard instrument (Thermo USA).

### 2.6. Statistical Analysis

The data were analyzed using SPSS for Windows, Version 18.0 (SPSS Inc., Chicago, IL, USA). The data are shown as mean ± standard error of the mean (SEM). Before statistical analysis, parameters without a normal distribution were logarithmically transformed to approximate the normal distribution. Single factor analysis of variance was used to compare measurement indexes among multiple groups. *χ*^2^-test or Fisher's exact test was used for the analysis of categorical variables. Multiple stepwise regression analysis was used in the multivariate analysis, with vaspin as dependent variable, and gender, age, duration of diabetes, BMI, waist-to-hip ratio (WHR), systolic blood pressure (SBP), hs-CRP, TG, TC, LDL-C, HDL-C, FPG, FINS, HOMA-IR, mass of visceral adipose, and HbA1C as independent variables. A *P* value of <0.05 was considered statistically significant.

## 3. Results

### 3.1. Baseline Clinical and Biochemical Characteristics of Participants

The baseline characteristics and biochemical characteristics of the 150 patients in the T2DM group and 77 individuals in the control subgroups are shown in Table 1. Age, gender distribution, and diastolic blood pressure (DBP) were similar in the normal weight control (NWC), obese control (OC), and T2DM subgroups. However, BMI, WHR, and SBP were significantly higher in the OC and T2DM groups than in the NWC group (*P* < 0.05). These parameters and disease conditions were similar between the OC and T2DM groups (*P* > 0.05). BMI was significantly higher in the OC group than in the T2DM group (*P* < 0.05).

The FPG, plasma insulin, plasma C-peptide, HbA1c, mass of visceral adipose, and hs-CRP levels as well as the HOMA-IR were the highest in the T2DM group. The levels were higher in the T2DM group than in the NWC and OC groups (*P* < 0.05, for both). The concentrations of TC and LDL-C were similar between the T2DM and OC groups and were significantly higher than those in the NWC group (*P* < 0.05). The concentration of TG was the highest in the OC group (*P* = 0.041).

The serum vaspin concentration differed significantly among the NWC, OC, and T2DM groups (*F* = 12.76, *P* = 0.009). It was remarkably higher in the T2DM group than in the control groups (*P* < 0.01) and significantly higher in the OC group than in the NWC group (*P* < 0.05).

### 3.2. Baseline Clinical and Biochemical Characteristics of the T2DM Subgroups

The baseline characteristics and biochemical characteristics of the 150 patients in the three T2DM subgroups are shown in Table 2. Age, gender distribution, duration of diabetes, systolic blood pressure (SBP), and diastolic blood pressure (DBP) were similar among the normal weight diabetes (NWD), normal weight control (OWD), and obese diabetes (OD) groups. However, BMI and WHR were significantly higher in the OD group (*P* < 0.05), as expected. The WHR was similar between the NWD and OWD groups (*P* > 0.05), whereas the BMI was higher in the OWD group in the NWD group (*P* < 0.05).

The levels of FPG, plasma insulin, plasma C-peptide, hs-CRP, and mass of visceral adipose as well as the HOMA-IR were higher in the OD group than in the other two subgroups (*P* < 0.05, for both). The concentrations of TC, LDL-C, HbA1c, and TG were similar among the three subgroups, and the concentration of HDL-C was the highest in the NWD group (*P* = 0.045).

The serum vaspin concentration differed significantly among the three groups (*F* = 9.42, *P* = 0.023). It was significantly higher in the OD group than in the NWD and OWD groups (*P* < 0.05), whereas it was similar between the NWD and OWD groups (*P* > 0.05).

### 3.3. Baseline Clinical and Biochemical Characteristics of the Normal Weight Diabetes Group and Normal Weight Control Group

The baseline and biochemical characteristics of the NWD group and NWC group are shown in Table 3. Age, gender distribution, SBP, DBP, BMI, and WHR were similar between the two subgroups (*P* > 0.05). The FPG, plasma insulin, plasma C-peptide, HbA1c, hs-CRP, and mass of visceral adipose as well as the HOMA-IR were higher in the NWD than in the NWC (*P* < 0.05) group. The concentrations of TC, LDL-C, and TG were similar between the two subgroups, and the concentration of HDL-C was higher in the NWD subgroup (*P* = 0.046).

The serum vaspin concentration differed significantly between the two groups (*T* = 12.21, *P* = 0.022). It was significantly higher in the NWD subgroup than in the NWC subgroup (*P* < 0.05).

### 3.4. Analysis of Correlations between Vaspin Levels and Other Characteristics

Significant positive correlations between the serum vaspin concentration and BMI (*r* = 0.76, *P* = 0.024), WHR (*r* = 0.78, *P* = 0.019), TG (*r* = 0.57, *P* = 0.044), and HOMA-IR (*r* = 0.79, *P* = 0.018) were found in the control participants (NWC and OC groups combined). We also found significant positive correlations between the serum vaspin concentration and BMI (*r* = 0.75, *P* = 0.035), WHR (*r* = 0.79, *P* = 0.032), FINS (*r* = 0.56, *P* = 0.045), C-peptide (*r* = 0.58, *P* = 0.043), mass of visceral adipose (*r* = 0.83, *P* = 0.024), and HOMA-IR (*r* = 0.78, *P* = 0.033) among patients with T2DM (Table 3). The serum vaspin concentration did not correlate with glycemic measurements, including FPG and HbA1c, in either the control participants or T2DM patients (Table 4).

### 3.5. Multiple Stepwise Regression Analysis of Associations between Vaspin Concentration and Other Characteristics

Multiple stepwise regression analysis was applied to the data from all T2DM patients. With vaspin as dependent variable and gender, age, duration of diabetes, BMI, WHR, SBP, hsCRP, TG, TC, LDL-C, HDL-C, FPG, FINS, HOMA-IR, HbA1C, C-peptide, and mass of visceral adipose as independent variables, the analysis revealed a significant positive association between the serum concentration of vaspin and BMI, WHR, mass of visceral adipose, and HOMA-IR (*P* = 0.043, 0.037, 0.029, and 0.014, resp.; Table 5).

## 4. Discussion

Vaspin was proposed as interesting insulin-sensitizing and insulin mimetic adipokine [5]. Our findings confirm that vaspin levels are affected by both T2DM and obesity in older adults. In this study, we found that the concentration of vaspin was higher in obese individuals than in those with normal weight. In addition, the concentration of vaspin was higher in T2DM patients than in the control participants. The concentration of vaspin in normal weight T2DM was higher than in healthy control group with normal weight. Finally, the obese T2DM patients had higher serum vaspin concentrations than the normal weight or overweight T2DM patients.

According to the most recent surveillance data, the prevalence of diabetes in the elderly Chinese population (over 60 years) varies from 19.6% to 23% [13]. Aging is considered a biological process characterized by progressive deterioration of physiological functions and metabolic processes. Diabetes in older adults is linked to higher mortality, reduced functional status, and increased risk of institutionalization. Adiposity is one of the most common comorbidities, and visceral adiposity specifically is a strong, independent predictor of dyslipidemia [14] and insulin resistance [15]. Also, changes in visceral fat are associated with concomitant changes in glucose tolerance and insulin resistance [16]. It was reported that central adiposity bestows enhanced risk of metabolic complications as compared with peripheral adiposity [17]. Previous study has found that vaspin may improve the metabolism of glucose and decrease glucose concentration [18]. In our study, BMI, WHR, mass of visceral adipose, and HOMA-IR were higher in the OD group than in the NWD and OWD groups. These results are consistent with the fact that T2DM is characterized by impaired insulin secretion and varying degrees of insulin resistance. Youn et al. also found that patients with obesity or abnormal insulin sensitivity had elevated serum vaspin levels [19]. Additionally, the serum vaspin concentration in obese patients was higher than that in nonobese patients in the same research [17]. Salama et al. (2015) found that vaspin levels are increased in obese children and identified correlations between vaspin concentration and WHR; however, they concluded that vaspin was a less sensitive indicator of abdominal obesity and insulin resistance in obese children compared with visfatin [20]. Cekmez et al. reported that large for gestational age children had a higher vaspin concentration than those who were appropriately sized for gestational age [21]. However, other reports found no correlation between vaspin concentration and BMI [22, 23]. Consistent with many of these studies, our result showed that the serum vaspin concentration was significantly higher in T2DM patients than in control individuals (*P* < 0.05). In addition, among the control participants, the serum vaspin concentration was higher in the obese group than in the normal weight group. In our study, single factor correlation analysis of data for the control individuals revealed a positive correlation between the serum vaspin concentration and BMI and WHR, which further indicates that serum vaspin is related to obesity.

We also found that the serum vaspin concentration was positively correlated with HOMA-IR (*r* = 0.750, *P* < 0.05), which is consistent with earlier reports. These results suggest that insulin resistance is associated with high concentrations of vaspin, and vaspin may be involved in the development of insulin resistance in elderly patients. It is possible that vaspin plays a compensatory role, which may help delay the progression towards insulin resistance and diabetes in elderly patients to a certain extent.

As adiposity plays an important role in the development of diabetes in older adult, we analyzed the relationship between the serum vaspin concentration and adiposity in diabetes patients. In a study population of nonelderly diabetes patients, Dai et al. (2016) found that serum vaspin concentrations were elevated in T2DM patients [24] and that serum vaspin concentrations were higher in obese T2DM patients than in lean T2DM patients and nondiabetic obese subjects. Yang et al. (2015) found that average serum vaspin levels were much higher in obese patients than in nonobese patients in both DM groups [25]. In our study, our results obtained in elderly patients were similar, with the serum vaspin concentration being significantly higher in the OD group than in the NWD and OWD groups (*P* < 0.05).

Our multiple stepwise regression analysis revealed that the serum vaspin concentration was influenced by BMI, WHR, mass of visceral adipose, and HOMA-IR, which suggests that serum vaspin may play a protective role in the development of diabetes in elderly people. The mechanisms of vaspin's actions remain obscure. Previous research demonstrated that vaspin can play inhibitory roles against inflammation in vascular smooth muscle [26]. A role of vaspin in the regulation of food intake also has been indicated [9]. Interesting, we noticed that the concentration of vaspin was similar between the normal weight and overweight groups of T2DM patients. A possible explanation is that BMI does not precisely reflect the measurement of visceral adiposity, and vaspin is produced in visceral fat. Although BMI is the most widely used and most practical clinical measure of adiposity in adults and children, it does not specifically reflect the gradual loss of lean mass and shift to central fat accumulation that occurs in older adults [27]. It was reported that the WHR or waist circumstance may be more precise than BMI at reflecting the degree of visceral adiposity in older adults [28].

It is important to note that the current study has some limitations. First, the sample size was not large. In addition, this was an epidemiologic study, capable of identifying correlations between variables and not causal relationships. Therefore, further experimental studies are required to elucidate the molecular mechanisms underlying the observed associations between the serum vaspin concentration and various metabolic parameters. Although the current study had sufficient power to detect significant associations between the serum vaspin concentration and various metabolic parameters in older adults with and without diabetes, further large-scale studies are required to gain more insight into the role of vaspin in T2DM.

In conclusion, the results of our study show that, just as in middle-age adults, the serum vaspin concentration correlates with adiposity, insulin resistance, and diabetes. Vaspin may have a beneficial effect on insulin resistance and T2DM. The higher concentration of vaspin in obese and T2DM patients could be related to a compensatory reaction to poor insulin sensitivity.

Our study put forward a hypothesis that serum vaspin may play an important role in insulin resistance, obesity, and T2DM. Further investigations are needed to understand the regulation of vaspin and its role in the development and progression of obesity and T2DM in the elderly. Given its cross-sectional design, this study alone cannot possibly establish a causal relationship. Further prospective studies with a large sample size are warranted to confirm the observed associations in our study.

## Figures and Tables

**Table 1:** Baseline characteristics and biochemical measurements of the NWC, OC, and T2DM groups.

Parameters	NWC	OC	DM	*F* value	*P* value
Gender (F/M)	28/10	27/11	115/35	0.73	NS
Age (years)	74.21 ± 4.22	75.21 ± 1.72	73.3 ± 6.22	0.92	NS
Duration (years)			16.26 ± 2.73		NS
BMI (kg/m^2^)	22.25 ± 2.13	30.12 ± 3.25	26.52 ± 2.46	8.76	0.028
WHR	0.84 ± 0.24	1.05 ± 0.24	0.93 ± 0.24	6.28	0.038
SBP (mmHg)	127 ± 9	137 ± 13	136 ± 16	5.56	0.042
DBP (mmHg)	70 ± 6	68 ± 6	69 ± 7	1.21	NS
FPG (mg/dl)	5.42 ± 4.20	6.16 ± 3.16	8.41 ± 1.20	7.381	0.034
FIN (mIU/ml)	12.7 ± 5.7	17.8 ± 49.1	22.7 ± 7.2	9.32	0.024
HOMA-IR	4.14 ± 3.18	7.97 ± 4.66	11.41 ± 2.13	10.23	0.013
HbA_1c_ (%)	6.07 ± 1.97	6.65 ± 2.17	8.58 ± 0.32	7.15	0.032
TC (mg/dl)	3.29 ± 1.15	4.35 ± 0.64	4.61 ± 1.63	4.53	0.035
LDL-C (mg/dl)	2.21 ± 0.75	2.39 ± 1.17	2.92 ± 0.93	1.23	0.218
HDL-C (mg/dl)	1.78 ± 0.45	1.43 ± 0.50	1.19 ± 0.59	5.24	0.043
TG (mg/dl)	1.94 ± 1.24	2.61 ± 1.64	2.42 ± 0.69	5.32	0.041
Hs-CRP (mg/dl)	2.15 ± 4.30	5.02 ± 6.86	7.31 ± 3.84	8.12	0.025
Mass of visceral adipose (cm^2^)	78.34 ± 21.23	104.12 ± 7.98	112.21 ± 11.21	9.32	0.021
C-peptide (nmol/l)	0.78 ± 1.23	0.89 ± 2.12	1.25 ± 2.76	5.32	0.038
Vaspin (ng/ml)	192.5 ± 45.2	327.6 ± 54.8	451.9 ± 32.6	12.76	0.009

Data are presented as mean ± SE. NWC: normal weight control; OC: obese control; NS: not significant; DM: diabetes mellitus; BMI: body mass index; WHR: waist-to-hip ratio; SBP: systolic blood pressure; DBP: diastolic blood pressure; FPG: fasting plasma glucose; FINS: fasting insulin; HOMA-IR: homeostasis model assessment of insulin resistance; HbA1c: glycosylated hemoglobin A1C; TC: total cholesterol; LDL-C: low-density lipoprotein-cholesterol; HDL-C: high-density lipoprotein cholesterol; TG: triglycerides; hs-CRP: high-sensitivity C-reactive protein.

**Table 2:** Baseline characteristics and biochemical properties of the three T2DM subgroups.

Parameters	NWD	OWD	OD	*F* value	*P* value
Gender (F/M)	42/13	38/14	31/12	0.76	NS
Age (years)	75.8 ± 2.2	78.5 ± 1.7	75.5 ± 1.2	0.91	NS
Duration (years)	15.4 ± 2.9	16.9 ± 3.1	16.2 ± 2.5	0.86	NS
BMI (kg/m^2^)	22.57 ± 3.91	27.10 ± 3.62	32.43 ± 2.14	9.32	0.025
WHR	0.85 ± 0.54	0.91 ± 0.42	1.09 ± 0.27	6.68	0.037
SBP (mmHg)	131 ± 9	134 ± 12	141 ± 10	1.59	NS
DBP (mmHg)	69 ± 6	71 ± 7	73 ± 9	1.51	NS
FPG (mg/dl)	7.72 ± 3.87	8.26 ± 3.43	8.71 ± 1.65	0.78	NS
FIN (mIU/ml)	14.92 ± 5.32	16.91 ± 9.12	26.71 ± 4.34	7.24	0.027
HOMA-IR	8.54 ± 17.12	10.02 ± 19.43	16.01 ± 11.23	12.85	0.018
HbA_1c_ (%)	8.13 ± 1.65	8.76 ± 1.65	8.92 ± 2.11	0.98	NS
TC (mg/dl)	4.54 ± 1.54	4.42 ± 1.07	4.86 ± 0.73	0.87	NS
LDL-C (mg/dl)	2.36 ± 0.87	2.65 ± 1.43	3.19 ± 0.89	0.97	NS
HDL-C (mg/dl)	1.39 ± 0.32	1.26 ± 0.64	1.12 ± 0.43	3.41	0.043
TG (mg/dl)	1.76 ± 1.43	1.80 ± 1.23	1.91 ± 0.43	1.32	NS
Hs-CRP (mg/dl)	6.12 ± 3.43	7.22 ± 5.32	9.54 ± 2.65	3.12	0.042
Mass of visceral adipose (cm^2^)	91.23 ± 21.34	102.32 ± 11.21	125.76 ± 32.12	9.87	0.022
C-peptide (nmol/L)	0.68 ± 1.12	0.79 ± 2.65	1.18 ± 2.12	8.32	0.028
Vaspin (ng/ml)	382.1 ± 21.3	406.6 ± 17.3	498.2 ± 17.1	10.21	0.021

NWD: normal weight diabetes; OWD: overweight diabetes; OD: obese diabetes; NS: not significant; DM: diabetes mellitus; BMI: body mass index; WHR: waist-to-hip ratio; SBP: systolic blood pressure; DBP: diastolic blood pressure; FPG: fasting plasma glucose; FINS: fasting insulin; HOMA-IR: homeostasis model assessment of insulin resistance; HbA1c: glycosylated hemoglobin A1C; TC: total cholesterol; LDL-C: low-density lipoprotein cholesterol; HDL-C: high-density lipoprotein cholesterol; TG: triglycerides; hs-CRP: high-sensitivity C-reactive protein.

**Table 3:** Baseline characteristics and biochemical properties of the NMW and NWC.

Parameters	NWD	NWC	*T* value	*P* value
Gender (F/M)	42/13	28/10	0.89	NS
Age (years)	75.8 ± 2.2	74.21 ± 4.22	1.23	NS
BMI (kg/m^2^)	22.57 ± 3.91	22.25 ± 2.13	1.32	NS
WHR	0.85 ± 0.54	0.84 ± 0.24	0.98	NS
SBP (mmHg)	131 ± 9	127 ± 9	1.21	NS
DBP (mmHg)	69 ± 6	70 ± 6	1.43	NS
FPG (mg/dl)	7.72 ± 3.87	5.42 ± 4.20	5.78	0.045
FIN (mIU/ml)	14.92 ± 5.32	12.7 ± 5.7	6.13	0.041
HOMA-IR	8.54 ± 17.12	4.14 ± 3.18	13.85	0.015
HbA_1c_ (%)	8.13 ± 1.65	6.07 ± 1.97	7.18	0.037
TC (mg/dl)	4.54 ± 1.54	3.29 ± 1.15	3.11	0.067
LDL-C (mg/dl)	2.36 ± 0.87	2.21 ± 0.75	1.07	NS
HDL-C (mg/dl)	1.39 ± 0.32	1.78 ± 0.45	4.11	0.046
TG (mg/dl)	1.76 ± 1.43	1.94 ± 1.24	1.32	NS
Hs-CRP (mg/dl)	6.12 ± 3.43	2.15 ± 4.30	11.12	0.022
Mass of visceral adipose (cm^2^)	91.23 ± 21.34	78.34 ± 21.23	5.87	0.042
C-peptide (nmol/L)	0.68 ± 1.12	0.78 ± 1.23	5.32	0.048
Vaspin (ng/ml)	382.1 ± 21.3	192.5 ± 45.2	12.21	0.020

NWD: normal weight diabetes; NWC: normal weight control; NS: not significant; DM: diabetes mellitus; BMI: body mass index; WHR: waist-to-hip ratio; SBP: systolic blood pressure; DBP: diastolic blood pressure; FPG: fasting plasma glucose; FINS: fasting insulin; HOMA-IR: homeostasis model assessment of insulin resistance; HbA1c: glycosylated hemoglobin A1C; TC: total cholesterol; LDL-C: low-density lipoprotein cholesterol; HDL-C: high-density lipoprotein cholesterol; TG: triglycerides; hs-CRP: high-sensitivity C-reactive protein.

**Table 4:** Pearson correlation analysis in control and T2DM groups.

Parameters	Control group		T2DM group	
	*R* value	*P*	*R* value	*P*
Age	0.24	0.354	0.02	0.789
BMI	0.76	0.024	0.75	0.035
WHR	0.78	0.019	0.79	0.032
TC	0.29	0.487	0.13	0.098
TG	0.57	0.044	0.36	0.146
LDL-C	0.23	0.487	0.41	0.123
HDL-C	0.55	0.072	0.56	0.086
HOMA-IR	0.79	0.018	0.78	0.033
FPG	0.28	0.416	0.22	0.239
FINS	0.47	0.075	0.56	0.045
HbA1c	0.29	0.408	0.13	0.231
C-peptide	0.51	0.065	0.58	0.043
Mass of visceral adipose	0.78	0.019	0.83	0.024

BMI: body mass index; WHR: waist-to-hip ratio; TC: total cholesterol; TG: triglycerides; LDL-C: low-density lipoprotein cholesterol; HDL-C: high-density lipoprotein cholesterol; HOMA-IR: homeostasis model assessment of insulin resistance; FPG: fasting plasma glucose; FINS: fasting insulin; HbA1c: glycosylated hemoglobin A1C.

**Table 5:** Risk factors of high vaspin concentration in patients with T2DM by multiple stepwise regression analysis.

Parameters	OR	95% CI	*P* value
BMI	1.545	1.342–1.742	0.043
WHR	1.625	1.435–1.812	0.037
HOMA-IR	1.943	1.712–2.254	0.014
Mass of visceral adipose	1.765	1.542–2.012	0.029

OR: odds ratio; 95% CI: 95% confidence interval; BMI: body mass index; WHR: waist-to-hip ratio; HOMA-IR: homeostasis model assessment of insulin resistance.
